# Influence of Environmental Conditions and Physical Modifications on the Gelation Behavior of Spent Brewers’ Yeast Protein

**DOI:** 10.3390/foods15213804

**Published:** 2026-10-23

**Authors:** Louisa Wötzel, Sarah Baumgartner, Sabina Paulik, Tomas Kurz, Mario Jekle

**Affiliations:** 1 Department of Plant-Based Foods, Institute of Food Science and Biotechnology, University of Hohenheim, 70599 Stuttgart, Germany; louisa.woetzel@uni-hohenheim.de (L.W.);; 2 ProteinDistillery GmbH, 73760 Ostfildern, Germany

**Keywords:** food proteins, alternative proteins, ultrasonication, thermal modification, single cell protein, *Saccharomyces cerevisiae*, gel strength, food hydrocolloids

## Abstract

Single cell protein from spent brewer’s yeast is a promising alternative food protein. However, its techno-functional properties, such as gelation, remain insufficiently characterized due to strong raw material dependency. This study investigated the gelation behavior of spent brewer’s yeast protein (YP) under various environmental conditions, as NaCl and pH. Moreover, the potential of modification approaches, such as ultrasonication and thermal treatment, to enhance gel strength and rupture force was evaluated. In addition, amino acid composition and solubility were assessed. Untreated YP gelling temperature at pH 7 was determined at 53.17 ± 0.20 °C. Gel strength was increased by 34% via 0.25 M NaCl addition at pH 7. Together with adjusting the YP concentration, this allows tailoring of the gelation behavior for specific food applications with these parameters. Furthermore, ultrasonication was identified as a suitable modification approach to increase the gelation properties. Ultrasonication treatment (5 min, 20 kHz, 73 W) resulted in a gel strength of 2.15 ± 0.03 N compared to 1.63 ± 0.06 N for untreated YP (15% concentration, pH 7). Overall, these findings provide novel insights into the modulation of YP gelation behavior via environmental factors and modification approaches and consequently about its application potential as a functional food ingredient.

## 1. Introduction

Single cell proteins (SCPs) are derived from microbial origins. Potential organisms used as SCP sources include algae, bacteria, and fungi. In the case of fungi, the yeast species *Candida* sp. and *Saccharomyces* sp. are commonly exploited for SCP extraction [1]. These proteins can be extracted from various industrial food side streams. This offers significant benefits considering resource-efficient waste management and sustainability [2]. One potential side stream is spent brewer’s yeast, the second largest by-product of beer production. Approximately two grams of spent brewer’s yeast result per liter of beer. This side stream can be upcycled to spent brewer’s yeast protein [3,4].

Commonly, spent brewer’s yeast contains over 85% water, whereby the remaining dry matter consists mainly of yeast cells and unwanted beer and trub solids [3,4,5]. The spent yeast cells commonly found in spent brewer’s yeast belong to the strain *Saccharomyces cerevisiae* and contain up to 60% protein. These proteins offer many advantages, such as a high essential amino acid content and a good biological value [6]. Furthermore, yeasts are overall known and common for human consumption and brewer’s yeast is generally recognized as safe (GRAS) by the FDA [3]. Additionally, SCPs derived from yeast can come along with lower land and water requirements, high growth rates, as well as smaller greenhouse gas emissions in comparison to animal-derived proteins, although the current infrastructure still needs further development. Moreover, since the need for a sufficient food supply is predicted to double until the year 2050, yeast proteins are an attractive option to help overcome future and present hurdles [7]. Furthermore, as yeast protein can be obtained from the side stream spent brewer’s yeast, its repurposed use as a raw material for SCP extraction could lead to waste reduction, support the development of a sustainable economy and reduce the environmental effect of beer brewing [4]. Thus, spent brewer’s yeast cells have great potential to be used for SCP extraction and as an environmentally attractive alternative protein raw material.

The transformation of spent brewer’s yeast into a valuable food protein requires a precisely controlled and adapted extraction procedure. Depending on this procedure, the obtained protein exhibits different visual, chemical, structural, and techno-functional features [8,9]. These features are relevant to evaluate the later application potential as a techno-functional food ingredient. Gelation is one key functionality that determines the suitability of SCPs for food applications. Protein functional properties can be strongly affected by extrinsic factors, such as pH and ionic strength, which may vary considerably depending on the intended food application [2]. Moreover, various modification approaches exhibit great potential to not only affect physicochemical characteristics, but also functional properties, such as gelation [2,9,10]. Thermal pre-treatment and ultrasonication represent two physical modification approaches that have already demonstrated high potential for functionality enhancement in other proteins [10,11,12]. According to Zink et al. [13], thermal treatment can be considered a physical approach, particularly when distinguishing physical from chemical modifications.

Protein gels’ viscoelastic properties are crucial for the development of texture and stability of alternative foods, as meat alternatives or cheese [2,8,14]. The gelation mechanisms of food proteins can be categorized into two main groups, heat- and cold-induced gelation. Both rely on heat-provoked protein unfolding, resulting in the exposure of formerly hidden amino acids. Subsequently, the formation of covalent and non-covalent protein–protein and protein–solvent interactions result in the formation of a three-dimensional gel network [8,14,15]. This heat- or cold-induced gel network formation is dependent on the protein’s intrinsic factors, including structure, molecular weight, and denaturation temperature. Extrinsic factors, as pH or ionic strength, may additionally influence protein network formation by altering intermolecular interactions and aggregation [2,8,16,17].

The main difference between heat- and cold-induced gelation is the temperature, at which the network formation occurs. During cold-induced gelation, network formation takes place after cooling and typically requires the addition of salt or pH-adjustment towards the isoelectric point. Heat-induced gel network formation already occurs at high temperatures and does not necessarily require the addition of ions. Still, cooling is often part of heat-induced gelation, as it can lead to further strengthening of the gel network [8,16,17]. The gelation mechanism and the properties of the resulting protein gels are very individual and, therefore, need to be investigated for each new protein source.

The available research on yeast protein implies that it exhibits potential as a techno-functional food ingredient, but that its gelation properties are either insufficiently studied or inferior compared to other protein sources [2,18,19,20]. However, since gelation is crucial for many foods, fundamental research focusing on the characterization as well as modification of yeast protein gelation properties is required. Comprehensive studies elucidating the impact of environmental conditions and physical modifications on the gelling properties of spent brewer’s yeast protein remain limited. Therefore, the impact of pH, NaCl addition and yeast protein concentration on the gelation behavior of spent brewer’s yeast protein was ascertained. Additionally, the effect of two physical modification approaches, ultrasonication and thermal treatment, was assessed. Analyses of amino acid composition, Zeta potential, and solubility complemented the findings about gelation properties. Overall, the results facilitate novel and fundamental insights for implementing spent brewer’s yeast as an alternative techno-functional protein ingredient for food applications. This will help overcome the current research gap of heat-induced gelation of spent brewer’s yeast proteins.

## 2. Materials and Methods

### 2.1. Materials

Spent brewer’s yeast protein product (YP) was provided by Protein Distillery (Ostfildern, Germany, one batch analyzed). The yeast cells belonged to spent brewer’s yeast from the strain *Saccharomyces cerevisiae* and were mechanically extracted. Further information about the extraction procedure was not available. The protein content (Kjeldahl, protein conversion factor of 5.5 based on Oliveira et al. [21]), water content, fats, and fibers were determined in an external lab based on § 64 LFGB L 06.00-6, L 06.00-3, L 06.00-7, and L 00.00-18 [22]. NaCl content was determined with an ICP-OES and amino acids with an LC-MS/MS. For all analyses, ultrapure double-distilled water with a conductivity of 0.055 μS/cm was used (referred to as H_2_O_bidest._). Throughout this study, the stated YP concentrations refer to the amount of YP powder used for the solution preparations rather than the actual protein content.

### 2.2. Zeta Potential

A 0.1% (*w*/*w*) YP stock solution was prepared using H_2_O_bidest_. under agitation at 500 rpm in a 20 °C water bath for 60 min. Following, aliquots of the stock solution were transferred into glass beakers and the pH was adjusted using 0.1 M NaOH and 0.1 M HCl to pH values between 3.1 and 8.3. The Zeta potential of YP solutions was measured using a Zetasizer Nano ZS equipped with an MPT-2 autotitrator (Malvern Panalytical, Malvern, UK) to determine the isoelectric point. The measurement was performed in triplicate, using three independently prepared samples.

### 2.3. Protein Solubility

The pH-dependency of the protein solubility was analyzed based on a modified Bradford method [23] using the QuickStart Bradford Protein Assay (BioRad Laboratories, Hercules, CA, USA). A 1% (*w*/*w*) YP stock solution was prepared using H_2_O_bidest_. under agitation at 500 rpm in a 20 °C water bath for 60 min. Afterwards, aliquots of the stock solution were transferred into glass beakers and the pH was adjusted using 0.1 M NaOH and 0.1 M HCl, between pH values of 3 and 8. The adjusted YP solution was stored overnight at 4 °C to ensure complete hydration. After storage and equilibration at room temperature, the samples were centrifuged at 10,000× *g* for 15 min (Laboratory centrifuge MiniSpin plus, Hamburg, Germany). The supernatants were filtrated using Chromafil RC-45/15 MS micro syringe filters (MACHEREY-NAGEL GmbH & Co. KG, Düren, Germany), with a pore size of 0.45 µm and regenerated cellulose as membrane material. The filtrate was diluted fivefold using H_2_O_bidest._. A standard calibration curve was prepared using the bovine serum albumin standards from the assay kit, with concentrations of 0.2–0.8 mg/mL. For the analysis, 100 µL of standard or sample were mixed with 5 mL of the Bradford reagent and incubated for 5 min. The absorption of this solution was photometrically measured at 595 nm. The YP solutions soluble protein content was determined based on the standard calibration curve (absorbance = 1.153 * concentration [mg/mL] + 0.1755, R^2^ = 0.994). The determined protein concentrations were corrected for the dilution factor of five to obtain the undiluted soluble protein concentration in mg/mL. The percentage solubility was subsequently calculated based on solubility [%] = (*c* * *V*/*m*) * 100%, where *c* represents the undiluted soluble protein concentration [mg/mL], *V* the sample volume (5 mL) and *m* the YP amount (50 mg). Solubility was therefore normalized to yeast protein powder mass. The measurements were performed in triplicate, using three independently prepared samples.

### 2.4. Rheological Analysis

The rheological analysis for the assessment of the gelling temperature were performed applying a modified method of Yang et al. [24]. The temperature sweep was conducted using a Rheometer MCR 302e (Anton Paar, Graz, Austria) in the linear viscoelastic range of the sample. A 15% (*w*/*w*) YP solution was prepared using H_2_O_bidest_. under agitation at 500 rpm for 60 min at 20 °C in a water bath. The pH was adjusted to pH 7 using 0.1 M NaOH. The coaxial cylinder geometry with a conical measurement cylinder (CC24, Anton Paar, Graz, Austria) and a gap width of 1 mm was filled with 11 mL of YP solution. The surface was covered with paraffin oil to prevent dehydration. The sample was subjected to a sinusoidal deformation under a constant frequency of 1 Hz and a constant strain of 1%. Temperature control was accomplished using a Peltier element and a water bath (Julabo 200 F, Anton Paar, Graz, Austria). The temperature sweep was conducted as follows: temperature was increased from 25 °C to 85 °C with a heating rate of 4.7 K/min, then held at 85 °C for 5 min and cooled down to 25 °C with a cooling rate of 4 K/min. The storage modulus G′ and the loss modulus G″ were acquired throughout the complete measurement. The intercept between G′ and G″ during heating yielded the gelling temperature. The measurements were performed in triplicate, using three independently prepared samples.

### 2.5. Yeast Protein Solutions Under Different Environmental Conditions

YP solutions were prepared under agitation at 500 rpm in a 20 °C water bath for 60 min. The pH value was adjusted using 0.1 M HCl and 0.1 M NaOH.

To analyze the impact of YP concentration, solutions with concentrations ranging between 5% and 20% (*w*/*w*) were prepared at pH 7. To examine the influence of NaCl, 15% (*w*/*w*) YP solutions were prepared at pH 7, followed by the addition of NaCl at nominal concentrations ranging between 0.25 M and 2 M NaCl. The required NaCl mass was calculated based on the initial volume of YP solution. The additional Na^+^ and Cl^−^ ions introduced during pH-adjustment were not accounted for in the calculation of the nominal NaCl concentrations. The influence of pH was ascertained by preparing 15% (*w*/*w*) YP solutions in the pH range of pH 5 to pH 8 using H_2_O_bidest_.

Five independently prepared replicates of each yeast protein solution were produced.

### 2.6. Physical Modifications of Yeast Protein Solutions by Ultrasonication and Thermal Treatment

The impact of ultrasonication and thermal treatment of 15% (*w*/*w*) yeast protein solutions at pH 7 on gelation behavior was ascertained. The respective YP solutions were prepared under agitation at 500 rpm in a 20 °C water bath for 60 min. The pH value was adjusted to pH 7 using 0.1 M HCl and 0.1 M NaOH.

Ultrasonication of 50 mL of YP solution was performed based on a modified version of Wötzel et al. [9] in a 100 mL beaker. An ultrasound sonicator (Sonopuls Ultraschall- Homogenisator HD 3100, Bandelin Electronics, Graz, Austria) with a 2 mm diameter sonotrode (MS 72, Bandelin Electronics) was used. Ultrasonication was operated in continuous mode at a frequency of 20 kHz and at a nominal power setting of 73 W. The sample was treated for either 1 min or 5 min and cooled using an ice bath throughout the treatment. This ensured that the solution temperature stayed at 20 °C.

For thermal treatment, 9 mL of YP solution was filled into 28 mm glass beakers and heated at 40 °C, 45 °C, 50 °C, 55 °C, and 60 °C for 15 min in a water bath. Subsequently, heat-treated samples were cooled to room temperature in a water bath for 15 min. The samples were kept inside the same glass beaker throughout thermal treatment, subsequently cooling and for the following heat-induced gelation and texture measurements.

Five independently prepared replicates of each treated yeast protein solution were produced.

### 2.7. Heat-Induced Gelation and Texture Measurement

Gels were prepared by transferring 9 mL YP solution from Section 2.5 and Section 2.6 into sealable glass beakers (28 mm diameter, 40 mm height). Subsequently, they were heated at 70 °C for 15 min in a water bath. Afterwards, the samples were cooled down to room temperature in a water bath for 15 min and stored in the fridge at 6 °C overnight (for 20–24 h). A gel from a 15% (*w*/*w*) YP solution at pH 7 was prepared analogues and served as a reference.

Prior to textural analyses, the gels were equilibrated to room temperatures in a water bath for 15 min. Gel strength and rupture force were analyzed applying a penetration test based on a modified version of Lee and Chung [25] at room temperature. A texture analyzer (TA.XT.plus Texture Analyzer, Stable Micro Systems, Godalming, UK) with a cylindrical probe (R/0.5, 12.7 mm diameter) was used. The gels were kept inside the glass beakers during the analysis. The penetration depth was set to 10 mm with a test speed of 1 mm/s and a trigger force of 0.01 N. Gel strength was defined as the force at maximum penetration. The rupture force was determined as the first identifiable peak (local maximum) of each individual force–distance curve (exemplarily indicated in Figure S1 of the Supplementary Materials). This local maximum served as an operational rupture force and was not automatically interpreted as evidence of macroscopic fracture.

The measurements were performed in quintuplicate, using five independently prepared/treated yeast protein solutions.

### 2.8. Data Analysis

Statistical analyses were performed using OriginLab (OriginPro, Version 2023. OriginLab Corporation, Northhampton, MA, USA). Statistical significance was determined using a one-way analysis of variance (ANOVA), followed by Tukey’s test for the mean comparison at a significance level of 0.05. Linear correlation analysis was conducted using the Pearson-correlation function of Origin at a significance level of 0.05.

## 3. Results and Discussion

### 3.1. Composition of the Yeast Protein

The spent brewer’s yeast protein product (YP) contained 76.4% protein (dry matter basis) and is thus considered a protein concentrate. Besides protein, it was composed of 9.1% fats (dry matter basis), 17.3% fiber (dry matter basis) and 0.14% NaCl (dry matter basis). The sum of compositional analytes slightly exceeds 100% on a dry matter basis, likely because of analytical variations, possible overlap between the measured fractions, and the indirect calculation of protein content from nitrogen. It should be noted that fibers were a main constituent of the YP, besides the actual protein content. Thus, rheological, and textural properties of the YP solution and gels cannot be solely attributed to the proteins itself. Fibers also have the potential to alter rheological and textural properties, for instance due to enhanced water holding capacity [2]. Moreover, protein fractions and subunits were not determined. These could provide further insights into the textural and rheological responses of YP.

The amino acid composition of YP was analyzed for 16 proteinogenic amino acids and is listed in Table 1, together with the amino acid requirements for an adult stated by the FAO and WHO [26]. The comparison with adult amino acid requirements is included only as a nutritional reference and should not be interpreted as a direct assessment of protein quality. Tryptophan, one of the nine essential amino acids for humans, was not determined in this study. The content of the remaining eight essential amino acids determined for YP fulfills and even exceeds the requirements in regard to human nutrition based on a mean total protein requirement for an adult of 0.66 g/kg per day [26]. Therefore, yeast protein derived from spent brewer’s yeast could be a promising alternative to animal protein sources, based on the determined eight essential amino acids. Consequently, yeasts and yeast proteins may be added to various foods to enhance their nutritional value [2]. However, this is based only on eight out of nine essential amino acids and thus does not provide a complete assessment of protein quality.

Besides the nutritional importance, the amino acid composition is also connected to the techno-functional properties of a protein. Solubility, surface-active properties and gelation behavior can be significantly influenced by the amount of individual amino acids [8]. Sulfurous amino acids play an important role in gel formation. The contained thiol-groups can be oxidized to disulfide bonds, which are crucial for both the formation and stabilization of a gel network [27]. Moreover, hydrophobic amino acids, as leucin, can strongly impact the specific gel structure. Although individual amino acids might provide insight into the potential gelation behavior of proteins, the overall amino composition must be considered, as the gelation behavior cannot be hypothesized from individual amino acids [28].

### 3.2. Zeta Potential and Solubility

**Figure 1:**
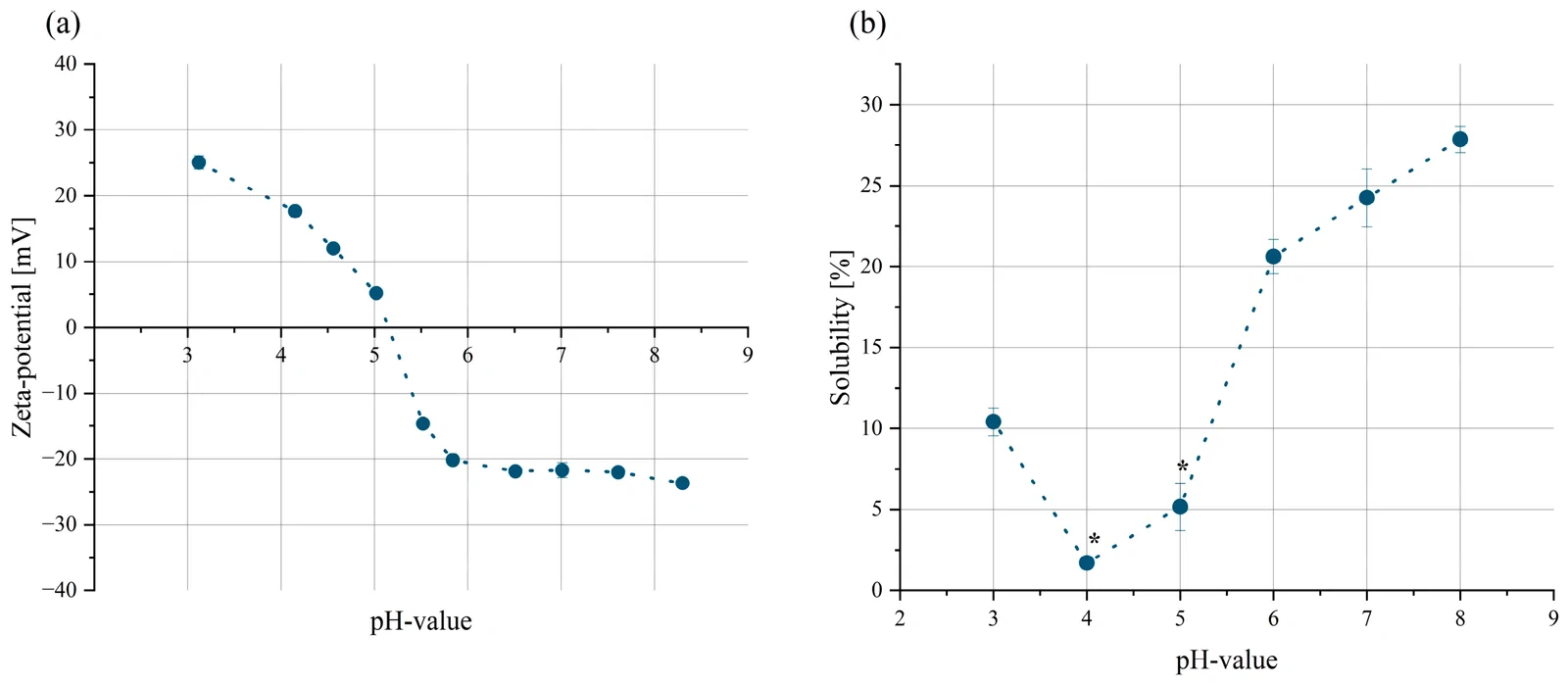
Zeta potential of a 0.1% (*w*/*w*) yeast protein solution (**a**) and solubility of a 1% (*w*/*w*) yeast protein solution (**b**) at different pH values. Values labeled with * fell below the Bradford calibration range (0.2–0.8 mg/mL) and should therefore be considered estimates. Values are shown as mean ± standard deviation (n = 3).

The Zeta potential serves as a parameter to determine the isoelectric point (IEP) of proteins, as well as to assess their pH-dependent net charge. Both IEP and Zeta potential can strongly impact protein solubility and gelation. This is connected to the proteins net charge, as solubility and net charge are typically lowest around the IEP. This can favor unwanted aggregation and electrostatic interactions at this pH region. As a consequence, insoluble and aggregated proteins might exhibit limited availability to form a protein gel network [2,15]. Therefore, solubility, Zeta potential, and IEP are critical parameters to assess in relation to YP gelation behavior. The determined IEP of YP was in the range of pH 5 to pH 5.5 (see Figure 1). Thus, it was situated in the typical pH region reported for other microbial single cell proteins (SCP) [2]. Furthermore, for YP to compare with other common food proteins, a similar pH-dependency would be beneficial. This is fulfilled with YP being in the typical IEP range of common proteins exploited as a functional food ingredient, as egg white and whey protein (IEP of 4.5), pea protein (IEP of 4.7) and soy protein (IEP of 4.6) [29,30,31,32]. Differences in the determined IEP of YP compared to common proteins could be connected to variances in the analysis methods, product composition, individual amino acid contents, and protein structure [2,33].

In addition to the Zeta potential, the solubility of alternative proteins is a crucial factor in determining their suitability for use in specific food applications. Insoluble proteins tend to form aggregates that can sediment and thus, reduce the accessibility to act as a functional food ingredient. Moreover, sufficient solubility is typically a pre-requisite for protein gelation, as insoluble proteins can exhibit limited availability to form a three dimensional gel network [2,15]. The pH-dependent solubility curve of YP is presented in Figure 1. It should be noted that the determined protein concentrations at pH 4 and pH 5 were below the Bradford calibration range of 0.2–0.8 mg/mL after fivefold dilution. Thus, the corresponding calculated solubilities at these pH values can only be considered as unvalidated estimates and should be interpreted with caution. Consequently, these two values were not included in the statistical comparison. One replicate at pH 3 was assessed to be close enough to the calibration range (0.19 mg/mL) and thus included in the statistical assessment. The other replicates at pH 3 were above 0.2 mg/mL.

The solubilities at the values of pH 3, pH 6, pH 7, and pH 8 differed significantly (ANOVA, *p* < 0.05). The unvalidated values at pH 4 and pH 5 might indicate that YP solubility could be lowest at this pH region. This might limit the application potential of YP in food products exhibiting similar pH values. However, further validated solubility assessment would be required to prove this hypothesis.

Nevertheless, the estimated low solubilities of YP between pH 4 and pH 5 would align with values reported for other SCPs. Hong et al. [34] posited that protein products from *Arthrospira* sp. exhibit solubilities lower than 15% around pH values of 3–4. Differences in the solubility values compared to other SCPs could be likely connected to variations in the protein’s structural elements. Moreover, it could be connected to the determined Zeta potentials and the IEP, as the colloidal stability of a suspension is strongly connected to the Zeta potential [35,36]. The Zeta potential of YP in the IEP region ranged between −10 mV and +10 mV (see Figure 1). Thus the solution can be considered as “very unstable” around the IEP based on Bhattacharjee [36] and Cano-Sarmiento et al. [35]. If the net charge of a protein is reduced, electrostatic attraction is favored. This has the potential to enhance protein aggregation, which consequently reduces its solubility [2,8]. The estimated lower solubility of YP at pH 4 compared to pH 5 could be related to the YP’s NaCl content of 0.13%. The presence of NaCl likely altered the electrostatic interactions and thus, the solubility of YP. This might have shifted the solubility curve away from the isoelectric point [37]. Besides NaCl, other compositional constituents, as fibers, could have affected the determined values. Moreover, the limitation of the solubility values at pH 4 and pH 5, resulting from the Bradford calibration range, could be another potential reason for the discrepancy of lowest solubility and IEP. Furthermore, the Zeta potential only provides information about colloidal stability considering electrostatic repulsive forces. Nonetheless, besides electrostatic interactions, colloidal stability is additionally affected by van der Waals forces. Thus, a protein suspension can be stable and soluble despite a differently indicating Zeta potential [36].

### 3.3. Rheological Properties of Yeast Protein and Gelation Behavior at Different Protein Concentrations

**Figure 2:**
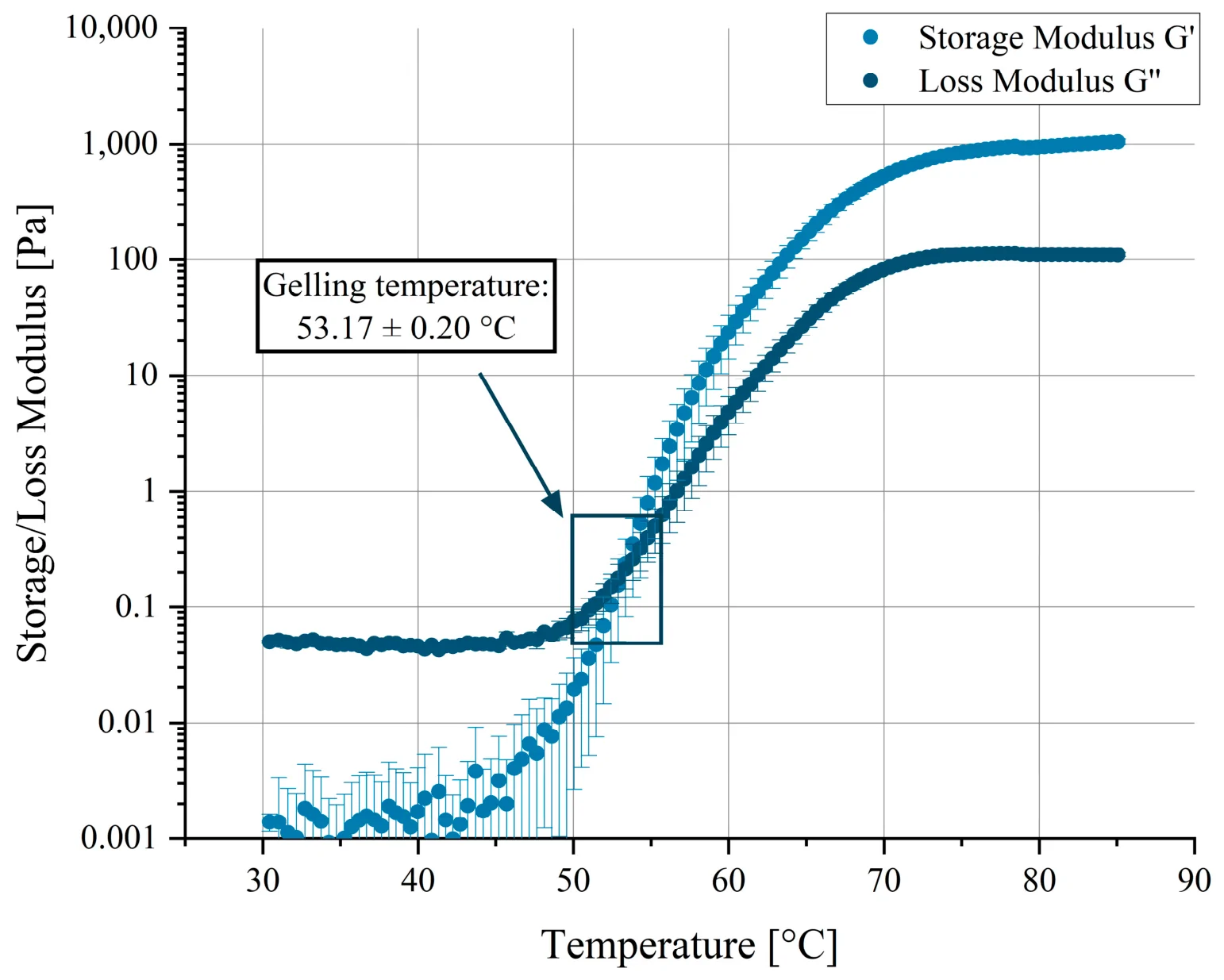
Storage and loss modulus of a 15% (*w*/*w*) yeast protein solution at pH 7 under sinusoidal deformation during heating and a constant frequency and strain of 1 Hz and 1%. Values are shown as mean ± standard deviation (n = 3).

The temperature-dependent rheological behavior of YP is illustrated in Figure 2. The gelling temperature was determined as the intersection point after which the elastic component (storage modulus G′) exceed the viscous component (loss modulus G″) [38]. This intersection was at a temperature of 53.17 ± 0.20 °C under the tested conditions and YP concentration of 15% (*w*/*w*). Thus, this temperature represents an operational gelling temperature. This determined gelling temperature aligns with the gelling temperatures reported for other SCPs. Grossmann et al. [39] determined a gelling temperature of 61 °C for protein from *Chlorella* sp. Moreover, *Spirulina* sp. proteins exhibited gelling temperatures between 40 °C and 60 °C, depending on the used protein concentrations [40]. The gelation temperature of YP was lower compared to plant-based proteins as soy and pea, which typically exhibit gelling temperatures over 70 °C [41,42]. Thus, YP exhibited a higher sensitivity to structural alterations induced at lower heating temperatures in contrast to plant proteins under the tested conditions. On the contrary, gelatin, an often-used animal-based gel forming biopolymer, has a lower gelling temperature with 33.6 °C, compared to YP [43]. Consequently, YP gelling temperature was found to be situated between that of conventional animal- and plant-based gelling agents, as well as within the range of other SCPs. This observation suggests that YP could serve as a potential alternative gelling agent in food products, based on its gelling temperature. Differences in YP gelling temperature compared to other common proteins could be related to variations in various sample specific intrinsic factors, as structural elements, composition or hydrophobicity [28].

Aside from the determination of the gelling temperature of YP, Figure 2 can provide information about the temperature range in which thermally induced rheological alterations occur [44]. Based on Strzelczak et al. [44], this temperature range, which resulted in thermally induced rheological changes in YP, may have begun at approximately 40–50 °C, as G′ demonstrated an increase between these temperatures. In addition, G′ stabilized at temperatures over 70 °C. This might indicate that further heating to 85 °C might not have exerted a significant influence on the rheological properties of the YP within this experimental framework. This resulted in the selection of 70 °C for the gel production temperature in this study.

**Figure 3:**
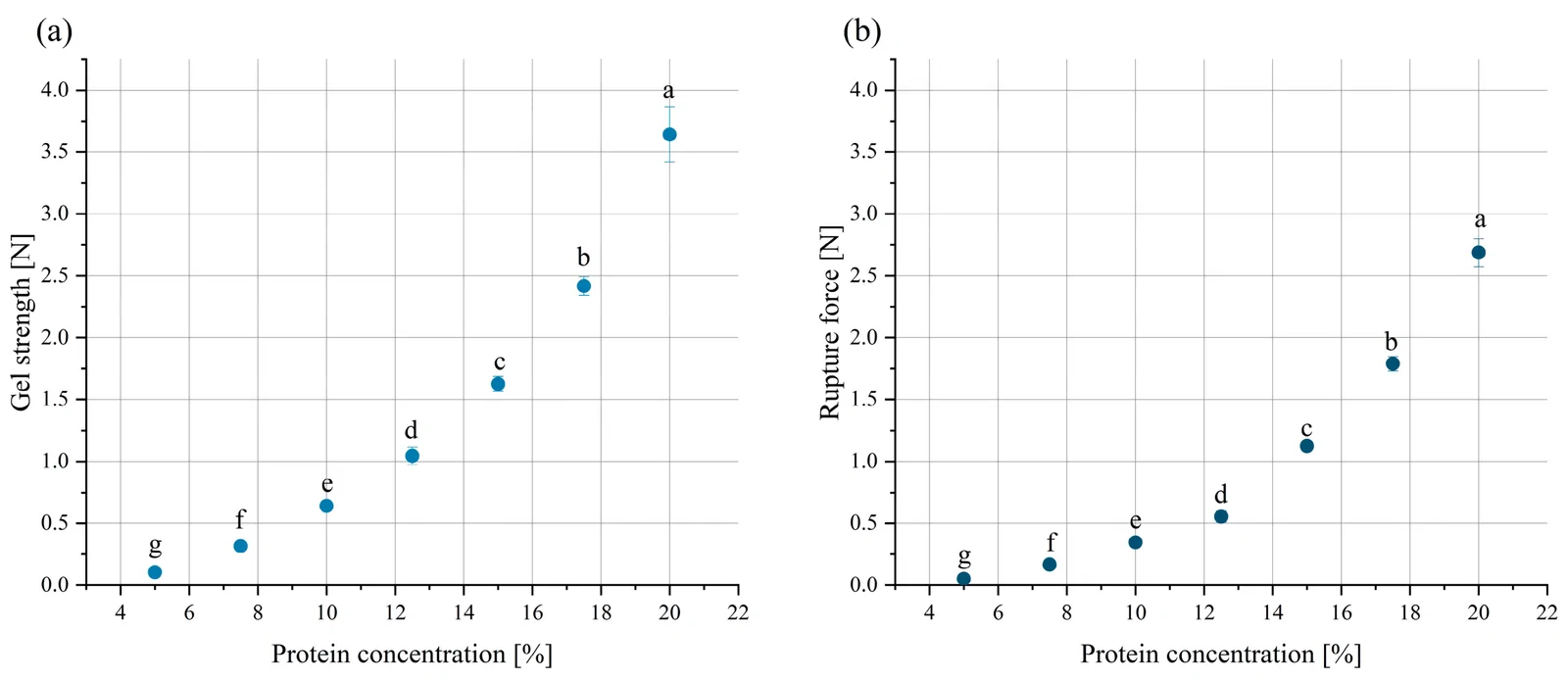
Gel strength (**a**) and gel rupture force (**b**) of yeast protein gels at pH 7, with varying yeast protein concentrations (in % *w*/*w*). Values are shown as mean ± standard deviation (n = 5). Values that do not share the same letter are significantly different (ANOVA, *p* < 0.05).

The impact of YP concentration on the gel strength and rupture force is depicted in Figure 3. The gel strength as well as the rupture force significantly positively correlated with the YP concentration, based on a linear Pearson-correlation analysis (r = 0.960 and r = 0.945, *p* < 0.05). Accordingly, gel strength and rupture force increased from 5% (*w*/*w*) YP concentration to 20% (*w*/*w*) YP concentration. A YP concentration of 5% (*w*/*w*) was determined as the lowest concentration demonstrated to gel. The ability to demonstrate a measurable gel formation was determined based on the force–distance curve of the samples (see Figure S1, Supplementary Materials), when a rupture force could be measured. At 5% (*w*/*w*) YP concentration, the gel strength and rupture force were comparably low with 0.11 ± 0.01 N and 0.05 ± 0.01 N, respectively. Nevertheless, YP’s lowest concentration demonstrated to gel was lower compared to that of common proteins as whey protein with 9% [45], pea protein with 20%, or soy protein with 15% [46]. However, a direct comparison with these reported literature values is limited, as the protein concentrations may be expressed in different bases. Moreover, experimental conditions used to assess gel formation differ between studies, as well as intrinsic protein characteristics, including structural components. Nevertheless, the results imply that YP might be a suitable alternative to common plant proteins, considering that low amounts of protein are required for gel formation.

Above a YP concentration of 20% (*w*/*w*), solidification of the YP solution and sufficient sample handling was not possible anymore. This suggests that a maximum possible YP concentration of 20% (*w*/*w*) can be achieved within the study’s experimental framework. This YP concentration resulted in a gel strength and rupture force of 3.64 ± 0.22 N and 2.69 ± 0.11 N, respectively. Moreover, the YP concentration of 15% resulted in a gel strength and rupture force of 1.63 ± 0.06 N and 1.12 ± 0.03 N. This concentration was selected for the other experiments, as it enabled the formation of a measurable gel for all analyses and parameter settings.

The increase in gel strength and rupture force with higher YP concentrations was likely resulting from enhanced protein sidechain interactions and junction points. Moreover, the higher amount of proteins could have also favored the entanglement of proteins and their respective sidechains [14,28]. This presumably facilitated the formation of stronger and more robust elastic gels. In comparison to common plant proteins, such as pea protein, YP gelation properties were comparable or even higher [47]. This indicates that YP is a highly promising gelling agent alternative to existing plant-based proteins. The results of gelation temperature and gelation properties in relation to the YP concentration highlight the potential of YP for future industrial food applications.

### 3.4. Gel Properties at Varying NaCl Concentrations and pH Values

**Figure 4:**
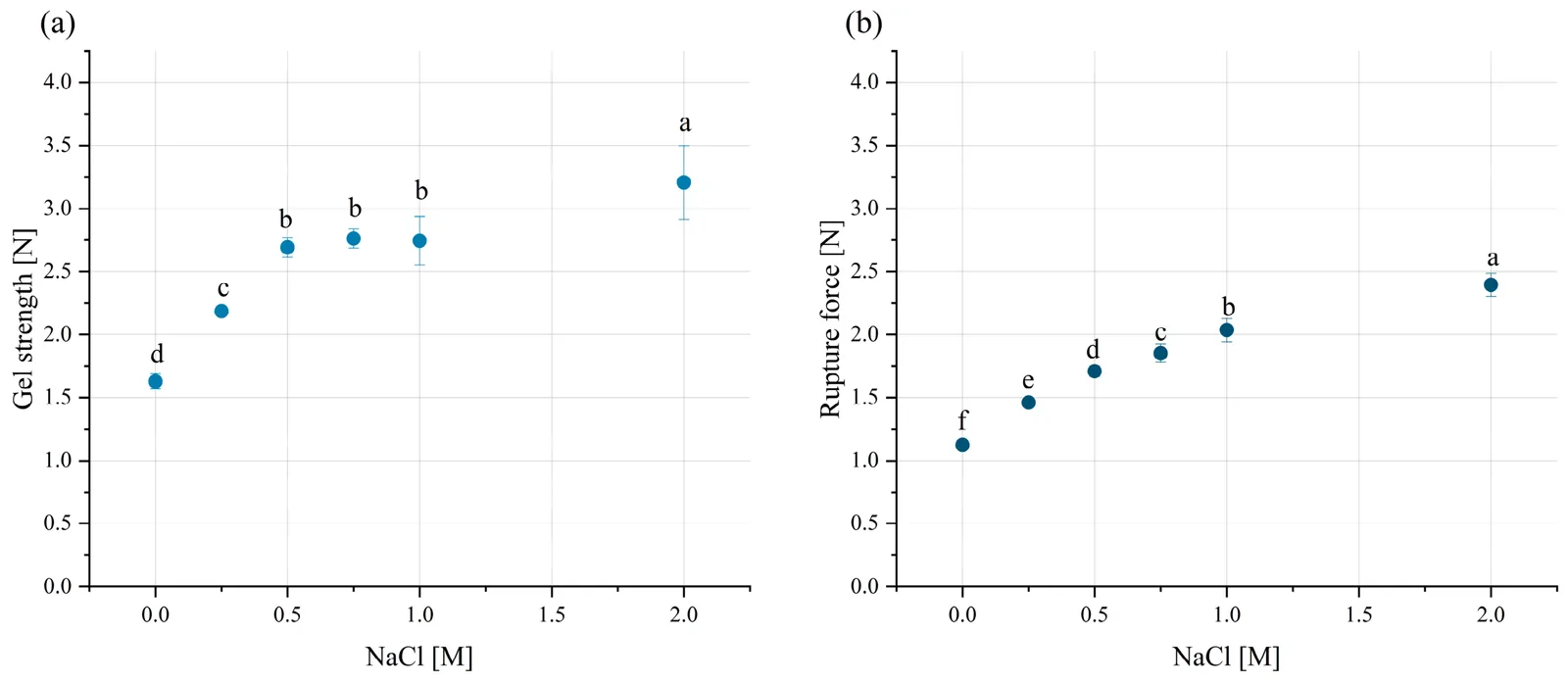
Gel strength (**a**) and gel rupture force (**b**) of yeast protein gels prepared from a 15% (*w*/*w*) solution at pH 7, with varying NaCl additions (0.25 M, 0.5 M, 0.75 M, 1 M, and 2 M, corresponding to 1.44, 2.83, 4.17, 5.48, and 10.47 g NaCl per 100 g of final yeast protein solution, respectively). Values are shown as mean ± standard deviation (n = 5). Values that do not share the same letter are significantly different (ANOVA, *p* < 0.05).

The gel strength and rupture force at different NaCl additions (0.25 M, 0.5 M, 0.75 M, 1 M, and 2 M) are shown in Figure 4. The NaCl additions correspond to approximately 1.44, 2.83, 4.17, 5.48, and 10.47 g NaCl per 100 g of final YP solution, respectively. These concentrations include the NaCl already present in the YP powder. The contribution of additional Na^+^ and Cl^−^ ions, introduced during pH-adjustment, to the overall NaCl concentration of the solutions cannot be excluded.

Both gel strength and rupture force significantly positively correlated with the NaCl addition, based on a linear Pearson-correlation analysis (r = 0.877 and r = 0.958, *p* < 0.05). Accordingly, all NaCl additions resulted in an increase in gel strength and rupture force. The gel strength increased from 1.63 ± 0.06 N to 3.21 ± 0.29 N at 2 M NaCl addition, reflecting a percentage increase of 97% in comparison to the reference gel with no NaCl addition. This increase in gelation properties could have been connected to enhanced electrostatic screening, which reduced the repulsive interactions of YP [8]. Consequently, protein–protein interactions might have been favored, which could have facilitated a stronger gel network formation. At high NaCl additions (over 1 M NaCl), a reduction in protein hydration and salting-out could have potentially further contributed to the formation of a strong and robust gel network [8,46,48]. Moreover, the NaCl addition might have also impacted the hydration and swelling behavior of the fibers present within the YP. This could have affected the gel network formation and possibly contributed to the observed textural changes in Figure 4 [49].

The effect of ionic strength on gel properties is typically strongly pH- and protein-dependent. Tanger et al. [48] determined that enhanced ionic strength reduced gelation properties of pea protein at pH 7. For soy protein gels, Chen et al. [50] observed that with increased NaCl addition (up to 0.5 M) the electrostatic repulsion was reduced. The stiffness of soy protein isolate gels, however, was not significantly influenced by NaCl incorporation. On the contrary, addition of NaCl to soy protein favored protein aggregation in another study [51]. Comparing the results for YP with the scarce research available on SCPs suggests protein-specific effects as well. For instance, Grossmann et al. [39] observed a reduction in gel strength of *Chlorella* sp. protein with NaCl addition.

The results for YP imply that its gelation properties are positively affected by NaCl addition. This is especially relevant for different food applications, as various foods often contain high amounts of salt, such as meat alternatives with up to 2.5 g per 100 g [52]. Thus, the improvement of gelling properties after 0.25 M and 0.5 M NaCl addition is relevant regarding food applications. Higher NaCl additions ranging from 0.75 M to 2 M, corresponding to approximately up to 10.47 g NaCl per 100 g of final YP solution, might however not be easily transferrable to foods. Still, the improvement at these higher NaCl additions provide valuable insights into the NaCl-dependent gelation behavior of YP.

**Figure 5:**
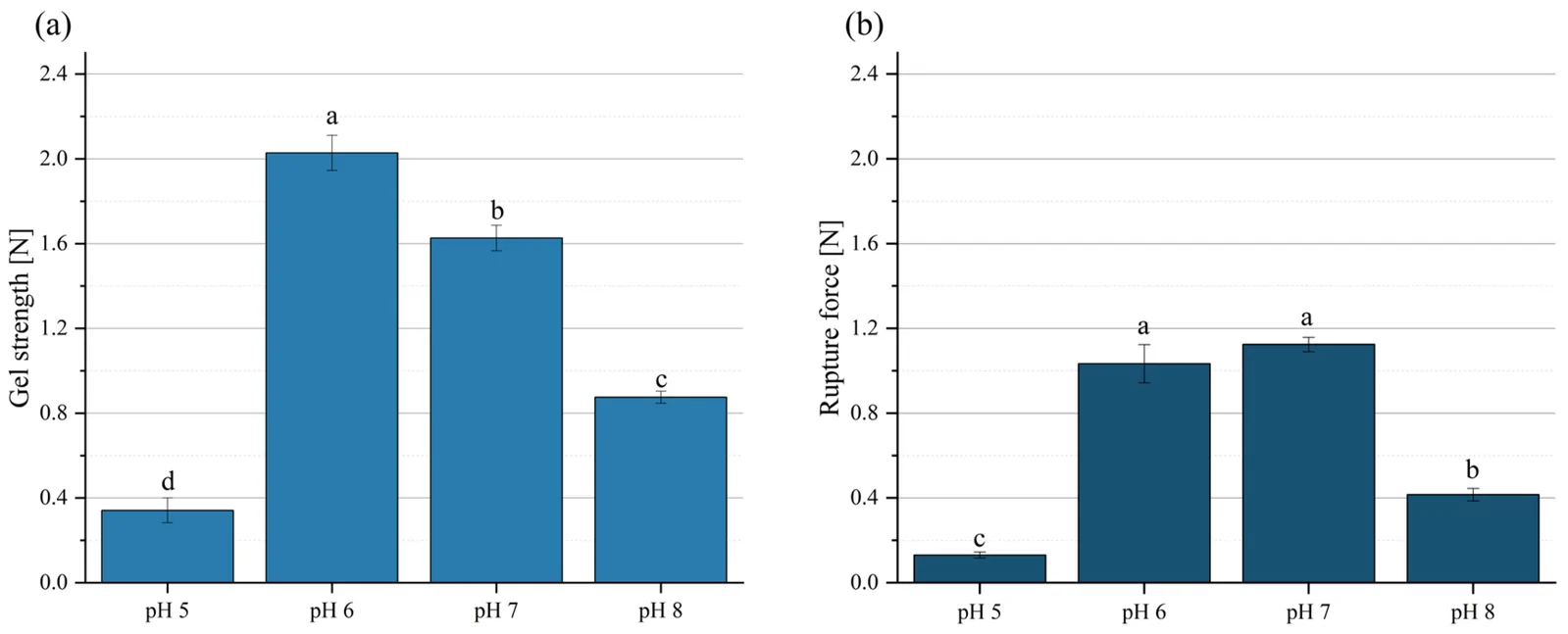
Gel strength (**a**) and gel rupture force (**b**) of yeast protein gels prepared from a 15% (*w*/*w*) solution at varying pH values. Values are shown as mean ± standard deviation (n = 5). Values that do not share the same letter are significantly different (ANOVA, *p* < 0.05).

In addition to NaCl, the pH value is another environmental factor that can affect the gelling behaviors of proteins [2]. Gel strength and rupture force of YP gels at pH values of 5, 6, 7, and 8 are shown in Figure 5. These pH values are of relevance, as a wide assortment of alternative food products typically fall within this pH range. The pH value of alternative burger patties, for instance, has been found to be at pH 5.8 [53]. The results for YP indicate that the gel strength varies significantly between pH 5 and pH 8, with a distinct optimum at pH 6 with 2.03 ± 0.08 N. No distinct pH optimum was observed for the rupture force, as it did not vary significantly between pHs 6 and 7. This might imply that the pH variation in pH 6 and 7 altered the gel matrix’s resistance to penetration, while the force required to induce initial operational rupture was not affected [25].

The lowest gel strength and rupture force were determined at pH 5 with 0.34 ± 0.06 N and 0.13 ± 0.01 N, respectively. This result aligns with those of the Zeta potential and estimated solubilities in Figure 1, as the creation of a gel network requires at least partial solubility of proteins, as posited by Volk, Wötzel et al. [2]. Shand et al. [54] and Volk, Wötzel et al. [2] also reported a strong pH-dependency of textural properties for pea protein and bacterial proteins. In contrast to YP, other SCPs, such as *Arthrospira platensis* protein, did not form a gel network at pH 5 [55]. Considering this, YP demonstrated potential to function as a gelling agent for slightly acidic food applications within the pH range where solubility and Zeta potential are limited, as compared to other SCPs.

The results of altering the environmental conditions, as NaCl addition (Figure 4) and pH values (Figure 5), revealed that NaCl addition exerted a positive effect on YP gelation properties. This observation is based on the maximum gel strength in Figure 4 being higher with 3.21 ± 0.29 N after 2 M NaCl addition, in contrast to the maximum gel strength of 2.03 ± 0.08 N at pH 6. However, the effects of environmental alterations can vary strongly depending on the protein source, due to various protein-specific intrinsic factors, such as electrostatic interactions, hydrophobicity, and amino acid composition [28]. Consequently, these observations are only applicable to the YP in this study. Shand et al. [54] observed that the positive effect on pea protein gels was more pronounced when the pH was altered, as opposed to by NaCl addition. This difference between pea and YP could be related to the maximum NaCl addition to the pea protein being 2% (*w*/*w*), which was lower than the 2 M NaCl, corresponding to ~10% NaCl addition to YP [54].

In conclusion, it was ascertained that the gel strength and rupture force of YP gels are significantly influenced by pH and NaCl alterations, which is essential for food applications with YP as a gelling agent. YP could function as a promising gelling agent in alternative meat products, as they commonly exhibit pH values near pH 6 [53] and thus close to the pH-dependent gel strength maximum. The reported positive effect of NaCl addition for YP was determined at pH 7. It could be a possibility that this effect might also occur at lower pH values and, thus, potentially counterbalance the weak gelling ability around the IEP.

### 3.5. Gel Properties After Physical Modifications

**Figure 6:**
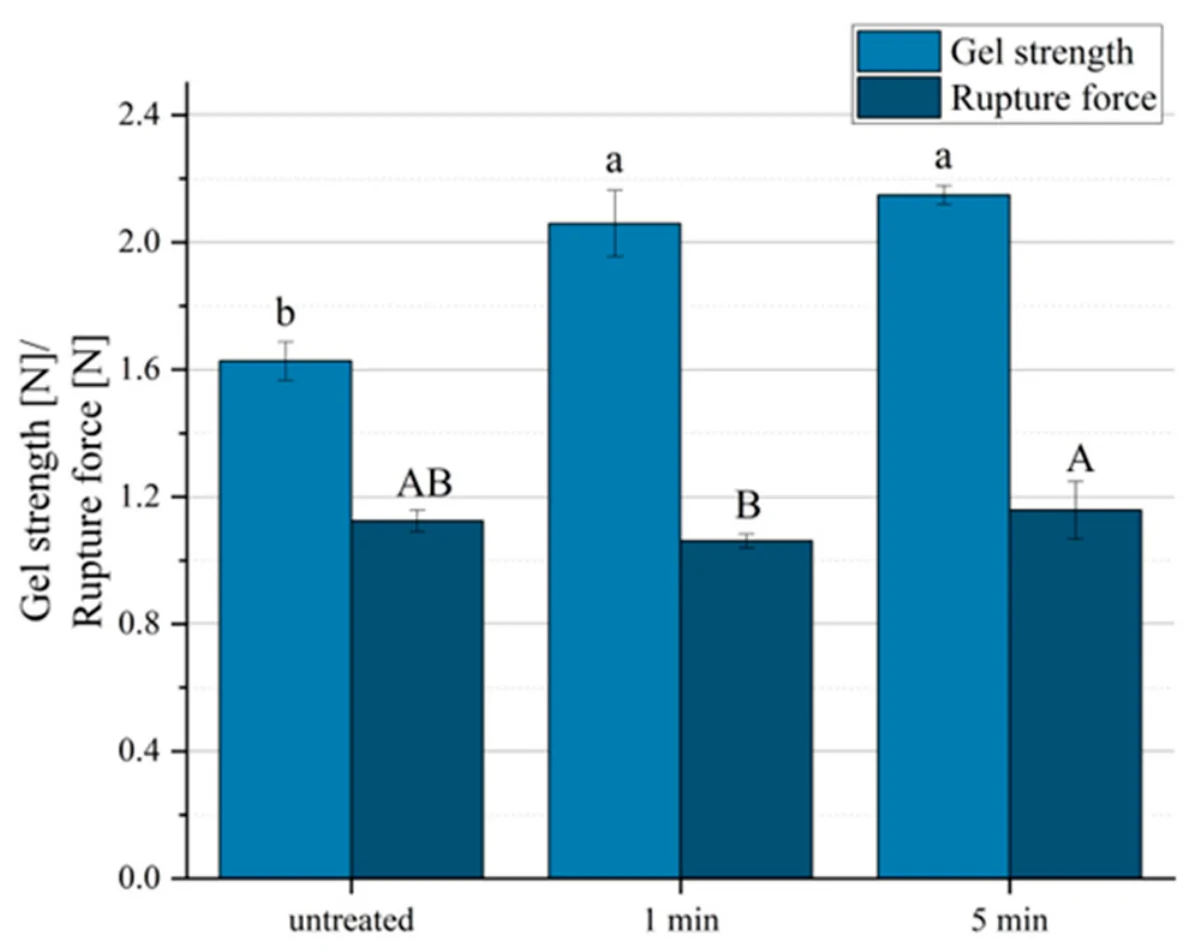
Gel strength and gel rupture force of yeast protein gels prepared from a 15% (*w*/*w*) solution at pH 7, modified via ultrasonication at 20 kHz and 73 W, for 1 min and 5 min. Reference gel was prepared from an untreated 15% (*w*/*w*) yeast protein solution at pH 7. Values are shown as mean ± standard deviation (n = 5). Different letters in the same parameter group (lowercase letters: gel strength; uppercase letters: rupture force) indicate significant differences (ANOVA, *p* < 0.05).

The influence of ultrasonication on the gelling properties of YP after two distinct treatment times (1 min and 5 min) is depicted in Figure 6. Ultrasonication increased the gel strength of the 15% (*w*/*w*) YP gel significantly from 1.63 ± 0.06 N for the untreated YP to 2.15 ± 0.03 N after 5 min of ultrasonication. The prolonged treatment time of 5 min did not additionally enhance the gel strength compared to the shorter treatment time of 1 min. Moreover, no significant effect of ultrasonication on the rupture force was observed compared to the untreated YP gel. During ultrasonication, gas bubbles are formed, expand, start to oscillate, and finally collapse as they cannot withstand the pressure anymore. Thus, high shear forces, mechanical and microbubble resonance, high pressures, temperature changes, and shock waves can be subjected to the ultrasonic treated samples [56,57]. The different effect on gel strength of the YP gels compared to rupture force might indicate that ultrasonication could have impacted the internal gel structure, thereby affecting the resistance to penetration. Conversely, the force required to initiate the first local operational gel rupture remained unaffected. Zhao et al. [10] and Wang et al. [11] posited that ultrasonication can alter the amount of hydrophobic junction zones and free SH groups, and decrease the particle size of proteins and aggregates. Moreover, it is discussed to potentially affect the secondary and tertiary structural elements of proteins, including the amount of α-helices and β-sheets, by unfolding or rearrangement [11]. These proposed structural alterations could have also occurred in the ultrasonicated YP and might have led to the enhanced gel strength after ultrasonication. This would align with functional and structural alterations reported for other protein sources. Zhao et al. [10] observed changes in secondary and tertiary structures of soy protein isolate, induced by ultrasonic treatment at 80 °C and 400 W for 5 and 15 min. Moreover, surface hydrophobicity and the amount of free sulfhydryl groups were increased, as well as gel strength and water holding capacity in the study of Zhao et al. [10]. In addition to alterations in the protein, Karim et al. [49] posited that ultrasonication exerts a potential effect on non-protein components of protein products, specifically fibers. Thus, ultrasonication might have induced alterations in the fiber structure and its hydration behavior, thereby potentially affecting the textural properties of the YP gels as well [49].

**Figure 7:**
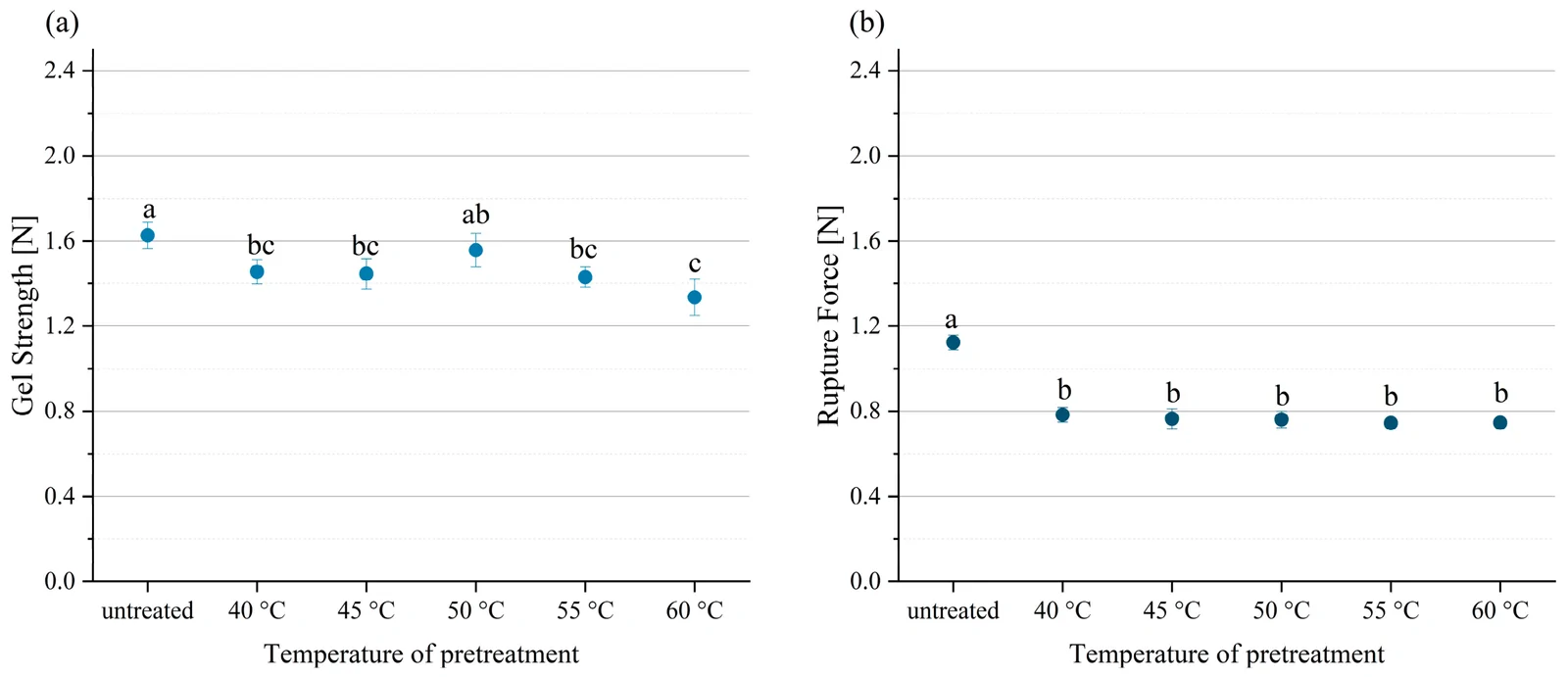
Gel strength (**a**) and gel rupture force (**b**) of yeast protein gels prepared from a 15% (*w*/*w*) solution at pH 7, pre-treated at different temperatures for 15 min. Reference gel was prepared from an untreated 15% (*w*/*w*) yeast protein solution at pH 7. Values are shown as mean ± standard deviation (n = 5). Values that do not share the same letter are significantly different (ANOVA, *p* < 0.05).

Thermal treatment of YP solutions presents another investigated physical modification approach. The resulting gel strength and rupture force after treatments between 40 °C and 60 °C are illustrated in Figure 7. Thermal treatments are known to potentially affect protein functionality. This is frequently associated with modifications of the secondary or tertiary structure of proteins, leading to unfolding and alterations in the proportion of structural elements, as a random coil or α-helices [10,13]. The temperature range of 40–60 °C was chosen based on the rheological assessment in Figure 2, as the heat-induced rheological changes started to occur at around 40 °C. The maximum treatment temperature of 60 °C was chosen to stay within the observed temperature range of rheological changes (Figure 2). Thermal treatments can break or change protein bonds, possibly resulting in a loss in the secondary and tertiary structure and resulting in an increased accessibility of formerly hidden functional groups. These groups may then start to realign and form new conformations, leading to changed functional properties and gelling behaviors [13,58]. Kong et al. [58] highlighted that the effect of thermal treatments on protein gelation properties can be either beneficial or lead to a reduction in these properties. The thermal treatment of YP reduced the gel strength and rupture force significantly, except for the gel strength at 50 °C treatment. For gel strength, the temperature treatment at 60 °C resulted in the maximum reduction from initially 1.63 ± 0.06 N to 1.34 ± 0.09 N. This loss in gel strength with higher temperatures could be connected to the temperature of 60 °C being above the determined operational gelation temperature of 53.17 ± 0.20 °C. Davis and Williams [59] posited that a specific temperature is required to induce irreversible structural rearrangements of proteins, as aggregation. These structural changes could have also occurred for the YP solutions that were heated above the determined gelling temperature of 53.17 ± 0.20 °C. This hypothesis would align with Sun and Arntfield [41], as they ascertained that the gelation properties of a plant protein isolate were significantly influenced by the thermal treatment as well. A higher heating rate decreased the gel strength and increased the gelation temperature of plant protein gels.

The reduction in rupture force of YP was not affected by variations in treatment temperature. The lowest temperature treatment at 40 °C resulted in a rupture force of 0.78 ± 0.03 N compared to 1.12 ± 0.03 N for the untreated YP. Consequently, the structural alterations occurring at 40 °C (see Figure 2) may already represent the most relevant changes for the rupture force.

In general, excessive heat treatment has the potential to result in uncontrollable aggregation and coagulation of proteins, instead of gel network formation [41,58]. This might explain the reduced gelation properties of the thermally treated YP gels. However, other functional properties of YP, such as foaming or emulsification, might be positively affected by thermal treatments. Adjonu et al. [60] depicted that heat pre-treatment of whey protein solutions at 80 °C for 15 min reduced the surface hydrophobicity. Shao et al. [61] determined that heating of soy protein isolates and concentrates reduced their viscosity and surface tension. Further research on YPs could ascertain the effect of protein type, longer or shorter treatment times, or pH value on the gelling abilities and other functional properties. Under the experimental set-up of this study, thermal treatment did not enhance gelation properties but reduced them.

## 4. Conclusions

This study aimed to characterize and to identify the extent of effects caused by environmental and physical modifications on spent brewer’s yeast protein (YP) gelation properties. The operational gelation temperature of YP was found to be at 53.17 ± 0.20 °C, indicating its potential use in food applications as an alternative to conventional, well-studied proteins. Further studies should elucidate whether this determined gelation temperature could be influenced by variations in the YP concentration. The ability to form gels at pH 6 and pH 7 favors the application of YP in foods with a neutral pH, for example in alternative meat products.

Moreover, the results ascertained that the gel strength and rupture force of YP gels significantly depend on the protein concentration, NaCl addition as well as the pH value. This enables the control of the gel formation by extrinsic factors in food applications. Furthermore, YPs’ lowest concentration demonstrated to gel was 5% (*w*/*w*). This underscores the potential of YP as a promising alternative gelling agent to conventional proteins (plant- or animal-based) for food applications. Moreover, the results implied that the gel strength and rupture force of YP were effectively increased by NaCl addition under the tested conditions. This might be advantageous for various food applications. As a substantial amount of foods contain NaCl or other salts, its presence might be a contributing factor to gel formation. This may facilitate the achievement of the desired gel properties with reduced amounts of YP. Additionally, the NaCl tolerance could be particularly beneficial for meat alternatives, where relatively high salt levels are common and may promote, rather than impair, gel formation and texture development.

Furthermore, YP gelation properties were effectively influenced by physical modifications, as ultrasonication resulted in an increase in the gel strength by 32% compared to the reference. This highlights the substantial potential of ultrasonication as a modification approach. Additional research could further ascertain the effect of prolonged treatment times, the impact on molecular and rheological alterations and on further techno-functional properties of YP. Thermal treatment of YP reduced the gel strength and rupture force.

Future studies should analyze the combined effect of environmental changes and physical modifications. Longer ultrasonication treatment times at lower pH values could promote stronger gel network formation due to aggregate disruption. This could extend the use of YP for applications requiring high gel strengths at low pH values, such as dairy alternatives. Moreover, comparisons with other proteins from *Saccharomyces* sp. or other yeasts could elucidate whether the observed effects are only limited to the YP of this study or apply to other yeast protein products. Considering the high fiber content of the YP, further studies should focus on elucidating its contribution to the rheological and textural properties observed.

In summary, this study provides essential knowledge and information regarding the gelation properties of spent brewer’s yeast protein under various environmental conditions and physical modification approaches. This was assessed for the first time in this extent. The observed effects underscore the enormous potential of YP for alternative food applications and the importance of further research to fully assess the techno-functional properties.

## Figures and Tables

**Table 1:** Amino acid composition in g/100 g protein of the analyzed yeast protein and the corresponding adult amino acid requirements stated by the FAO and WHO.

	Amino Acid Content in g/100 g Protein	
Amino Acid	Yeast Protein	Amino Acid Requirements for Adults [26]
Lysine ^1^	9.3	4.5
Isoleucine ^1^	5.6	3.0
Leucine ^1^	8.4	5.9
Methionine ^1^	1.9	1.6
Threonine ^1^	5.4	2.3
Histidine ^1^	2.6	1.5
Tyrosine + Phenylalanine ^1^	4.3 + 5.1	3.8
Glutamate	11.7	-
Aspartate	11.6	-
Serine	5.6	-
Proline	4.1	-
Alanine	7.0	-
Glycine	4.7	-
Arginine	5.7	-
Valine ^1^	6.5	3.9

^1^ Essential amino acids [26].

## Data Availability

The original contributions presented in this study are included in the article/Supplementary Material. Further inquiries can be directed to the corresponding author.

## Supplementary Materials

The following supporting information can be downloaded at: https://www.mdpi.com/article/10.3390/foods15213804/s1. Figure S1: Force–distance curves of yeast protein gels with varying powder concentrations at pH 7 and indicated area for rupture force and gel strength determination; (a) 10–20% (*w*/*w*) powder concentration and (b) 5% and 7.5% (*w*/*w*) powder concentration. Values are shown as mean ± standard deviation (n = 5).

## Abbreviations

The following abbreviations are used in this manuscript:

YP	Spent brewer’s yeast protein
SCP	Single Cell Protein
ANOVA	One-way analysis of variance
NaCl	Sodium chloride
IEP	Isoelectric point
n	Number of repetitions
MLR	Baden-Württemberg Ministry of Food, Rural Areas and Consumer Protection
