# Supplementary material for: Influence of Environmental Conditions and Physical Modifications on the Gelation Behavior of Spent Brewers’ Yeast Protein
Source: Foods. 2026 Oct 23;15(21):3804. doi: 10.3390/foods15213804 (PMC13762920; doi:10.3390/foods15213804)
Supplement: Supplementary file 1 [file foods-15-03804-s001.zip › Figure_S1.pdf]

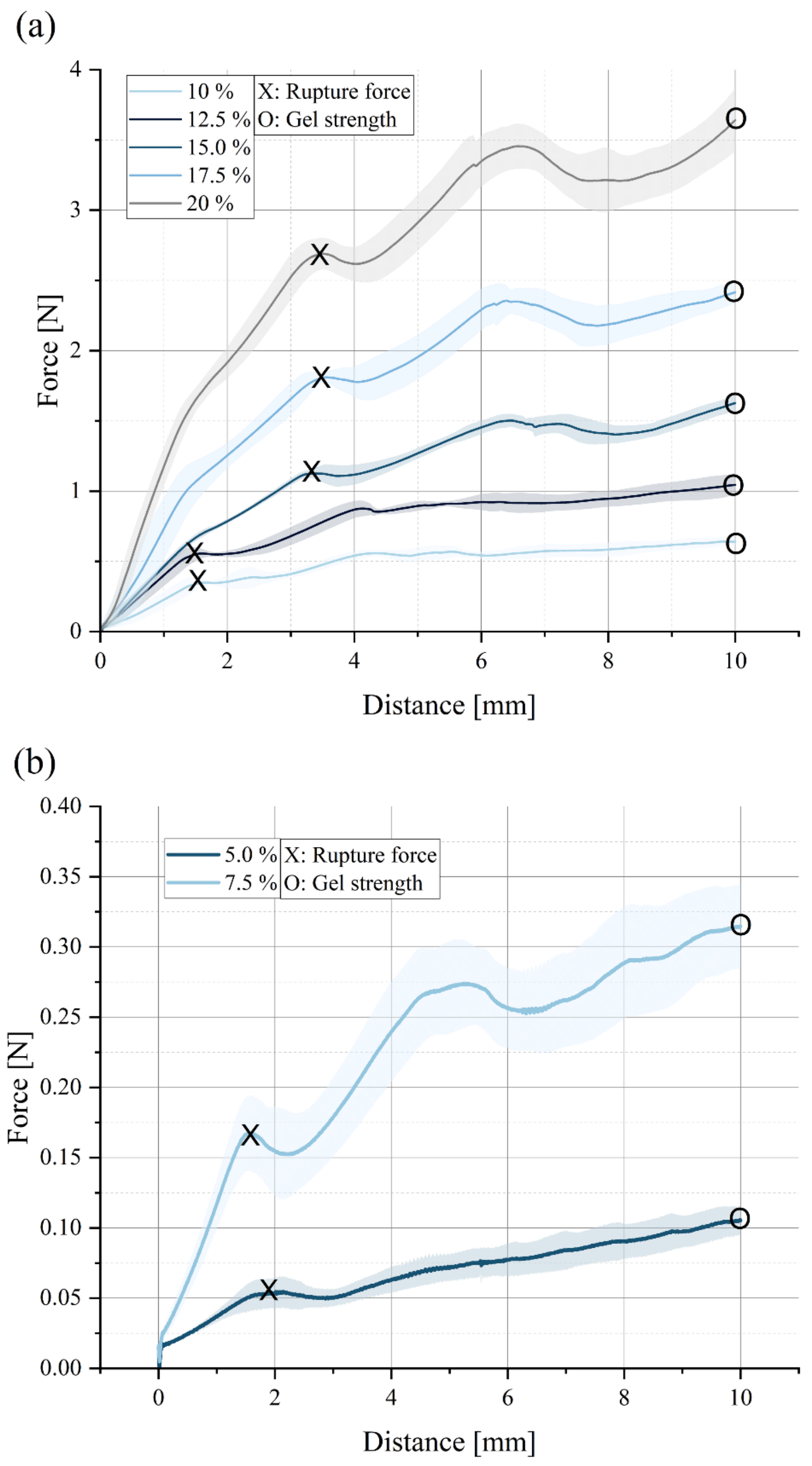

Figure S1. Force–distance curves of yeast protein gels with varying powder concentrations at pH 7 and indicated area for rupture force and gel strength determination; (**a**) 10–20% (*w*/*w*) powder concentration and (**b**) 5% and 7.5% (*w*/*w*) powder concentration. Values are shown as mean ± standard deviation (n = 5).
